# Efficacy of Citicoline Delivered via Brain Extracellular Space against Experimental Acute Ischemic Stroke in Rats

**DOI:** 10.7150/ijms.93482

**Published:** 2024-05-13

**Authors:** Guomei Zhao, He Chen, Junhao Yan, Zhiqian Tong, Yu Fu, Zhaoheng Xie, Hongbin Han

**Affiliations:** 1 Department of Geriatrics, Capital Medical University Affiliated Beijing Shijitan Hospital, Beijing 100038, China.; 2 Department of Radiology, China-Japan Friendship Hospital, Beijing 100029, China.; 3 Department of Human Anatomy, Histology and Embryology, School of Basic Medical Sciences, Peking University Health Science Center, Beijing, 100191, China.; 4 Beijing Key Laboratory of Magnetic Resonance Imaging Equipment and Technique, Beijing 100191, China.; 5 Oujiang Laboratory (Zhejiang Lab for Regenerative Medicine, Vision and Brain Health), Institute of Aging, Key Laboratory of Alzheimer's Disease of Zhejiang Province, Zhejiang Provincial Clinical Research Center for Mental Disorders, The Affiliated Wenzhou Kangning Hospital, School of Mental Health, Wenzhou Medical University, Wenzhou, Zhejiang, 325035, China.; 6 Department of Neurology, Peking University Third Hospital, Beijing 100191, China.; 7 Institute of Medical Technology, Peking University Health Science Center, Beijing 100191, China.; 8 Department of Radiology, Peking University Third Hospital, Beijing 100191, China.; 9 NMPA key Laboratory for Evaluation of Medical Imaging Equipment and Technique, Beijing 100191, China.

**Keywords:** citicoline, acute ischemic stroke, extracellular space

## Abstract

**Objective:** Citicoline can be used to reduce acute ischemic stroke injury via venous infusion, however, its protective effects in the brain extracellular space remain largely unknown. Herein, we investigated the brain protective effects of citicoline administered via the brain extracellular space and sought precise effective dosage range that can protect against ischemic injury after experimental ischemic stroke in rats.

**Methods**: Fifty-six Sprague-Dawley rats were randomly divided into control, intraperitoneal (IP), caudate-putamen (CPu)-25, CPu-40, CPu-50, CPu-60 and CPu-75 groups based on the infusion site and concentration of citicoline. Two hours after the administration of citicoline, the rats were subjected to a permanent middle cerebral artery occlusion to mimic acute ischemic stroke. Then, the brain infarct volume in rats after stroke was measured and their neurological deficiency was evaluated to explain the protective effects and effective dosage range of citicoline.

**Results:** Compared to the control and IP groups, brain infarct volume of rats in CPu-40, CPu-50, and CPu-60 groups is significant smaller. Furthermore, the brain infarct volume of rats in CPu-50 is the least.

**Conclusions:** Here, we showed that citicoline can decrease the brain infarct volume, thus protecting the brain from acute ischemic stroke injury. We also found that the appropriate effective citicoline dose delivered via the brain extracellular space is 50 mM. Our study provides novel insights into the precise treatment of acute ischemic stroke by citicoline via the brain extracellular space, further guiding the treatment of brain disease.

## Introduction

Stroke remains one of the most common causes of mortality and long-term disability, associated with onerous socioeconomic consequences 1. Citicoline is salient for membrane phospholipid biosynthesis and may enhance endogenous brain plasticity and repair, thus to decreasing acute brain damage and facilitating functional recovery in animal models of acute stroke 2. However, in case of acute ischemic stroke, the reduced volume fraction of brain extracellular space (ECS) and postischemic hypoperfusion before fast and successful recanalisation of the occluded artery reduces the infusion and uptake of citicoline in the ischemic area of the brain 3,4.

The brain ECS is defined as the space between neighboring neural cells in the brain, accounting for approximately 20% of the whole brain volume 5,6. The drainage of brain interstitial fluid (ISF) along the brain ECS deliver neurotransmitters and pharmaceutical agents in the brain 6,7. The simple diffusion delivery (SDD) technique allows the drugs diffused into the brain ECS along the brain ISF to bypass the blood-brain barrier, leading to high local drug concentrations without systemic exposure 4,8. In our previous study, we found that by using SDD, citicoline administered to the brain ECS prior to ischemic injury can result in substantially protective effects in a rat model of acute ischemic stroke 4. Nevertheless, the precise effective dosage range of citicoline for treating acute ischemic stroke remains unknown.

In the present study, we performed SDD to import citicoline or saline into the caudate putamen (CPu) or enterocoelia of rats. More specifically, rats were anesthetized and their right middle cerebral artery (MCA) was permanently occluded. Twelve hours after MCA occlusion, magnetic resonance imaging (MRI) was performed to obtain T1W and T2W images. Subsequently, the infarct volume of the rat brain was measured and compared between the groups. Eventually, our study provided conclusive evidence that 40-60 mM citicoline is the effective dose range for preventing acute ischemic stroke. Our findings provide novel insights into the precise treatment of acute ischemic stroke using citicoline via brain ECS.

## Materials and Methods

### Animal

All animal studies were conducted in accordance with the Peking University Biomedical Ethics Committee (permit number: LA201461), the National Guidelines for the Care and Use of Laboratory Animals and the Declaration of Helsinki. Rats were housed in individual cages under a 12 light/dark cycle and controlled temperature (22 ± 1 °C) and humidity (60 ± 5%), with food and water available *ad libitum*.

Fifty-six adult Sprague-Dawley rats (weighing 250-300 g) were anesthetized with pentobarbital sodium via IP injection and maintained at approximately 30 mg/kg/h. Subsequent experiments were performed when rats had lost their righting reflex. Rats were then randomly divided into seven groups. A heating pad was applied to maintain the rat body temperature at 37 ± 0.2 °C.

### Experimental groups and permanent MCA occlusion in rats

Anesthetized rats were fixed in a stereotaxic apparatus (Lab Standard Stereotaxic-Single; Stoelting Co., Wood Dale, IL, USA) with either of the following: 1) citicoline (5 µL, 25 mM) (CPu-25 group); 2) citicoline (5 µL, 40 mM) (CPu-40 group); 3) citicoline (5 µL, 50 mM) (CPu-50 group); 4) citicoline (5 µL, 60 mM) (CPu-60 group); 5) citicoline (5 µL, 75 mM) (CPu-75 group); or 6) 5 μL saline (control group) injected into the right CPu (AP: 0mm, L: -2.8 mm, V: -5.8 mm) as previously described 6,9,10. In the IP group, anesthetized rats received an IP injection of 2 g/kg citicoline.

Two hours later, the right middle cerebral artery (MCA) of rats was occluded as previously described 4,11. After surgery, rats were allowed to recover in their cages and soft mash was added to their diet.

Neurological evaluations were performed on all rats 30 min after MCA occlusion using a five-tiered grading system based on the Zea-Longa scale 4,12 as follows: 0, no neurological deficit; 1, failure to fully extend the left forepaw; 2, circling or walking to the left; 3, falling to the left; and 4, unable to walk spontaneously.

### MRI detection of MCA occlusion rats

**Figure 1:**
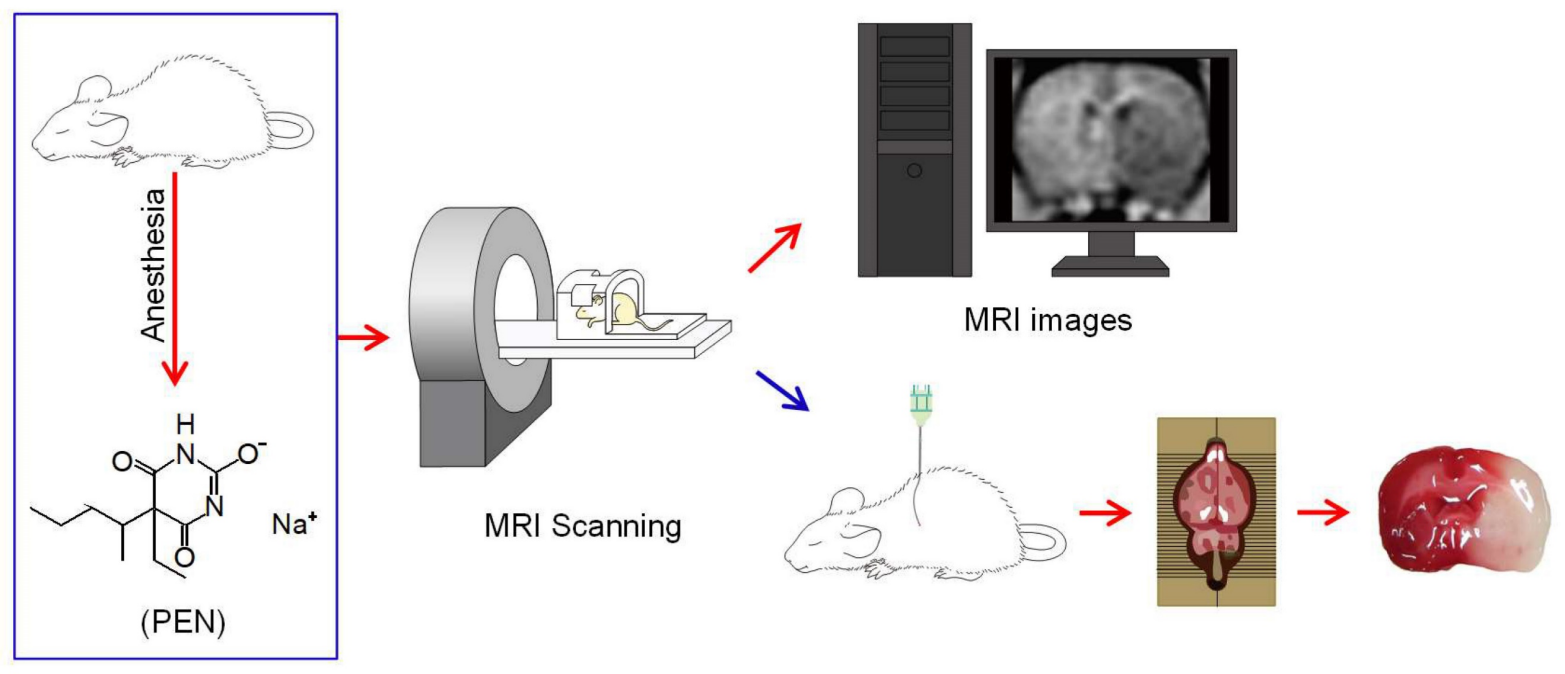
** Schematic diagram of the experiments.** All rats were anesthetized with pentobarbital sodium (PEN). Twelve hours after the MCA occlusion, rats were subjected to accept MRI detection. After the MRI, rats were euthanized and brain slices were collected.

Twelve hours after MCA occlusion, rats underwent MRI. A 3.0 T MR imaging system (Magnetom Trio, Siemens Medical Solutions, Erlangen, Germany) with an eight-channel wrist coil was used to obtain rat brain T1-weighted and T2-weighted MR images using a magnetization-prepared rapid acquisition with a gradient-echo (MP-RAGE) sequence (Figure 1). The acquisition parameters were set as previous described 4.

### Infarct volume determination in MCA occlusion rats

After performing the MRI, rats were euthanized sacrificed and brain slices were collected and stained with TTC (2,3,5-triphenyl-tetrazolium chloride, Sigma-Aldrich, St. Louis, MO, USA) as previously described 4 (Figure 1). The infarct volume of each brain slices was then analyzed and the infact regions were compared with those derived from MRI 4.

### Statistical analysis

The IBM SPSS software, (version 23.0; IBM Corp. Armonk, NY, USA) was used to analyze the data in the present study. One-way analysis of variance with Duncan's *post-hoc* test (overall significance level = 0.05) was used to compare data between different groups. Data are expressed as the mean ± standard error. Statistical significance was set at P < 0.05.

## Results

### Brain ECS administration of 40-60 mM citicoline significantly reduced brain infarct volume

**Figure 2:**
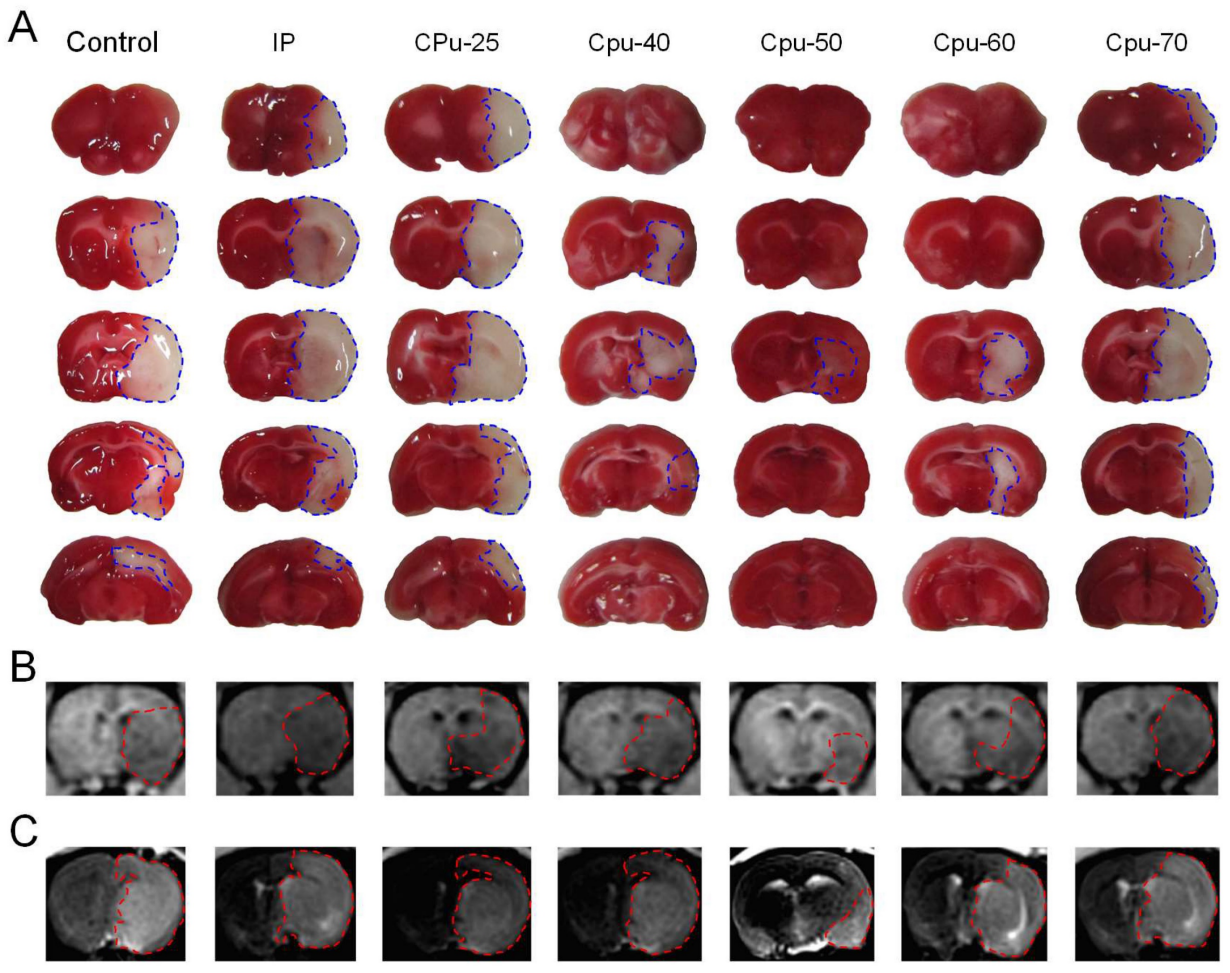
** Brain infarct area among different groups.** (A) The infarct area of brain slices among different groups. (B-C) MRI images of the brain infarct area among different groups.

We found that citicoline administered into rat brain ECS prior to MCA occlusion dramatically reduced the infarct volume compared with that in the control and IP group (Figure 2). We used MRI to define the size of brain infarct lesions and injury severity 4 (Figure 2).

**Figure 3:**
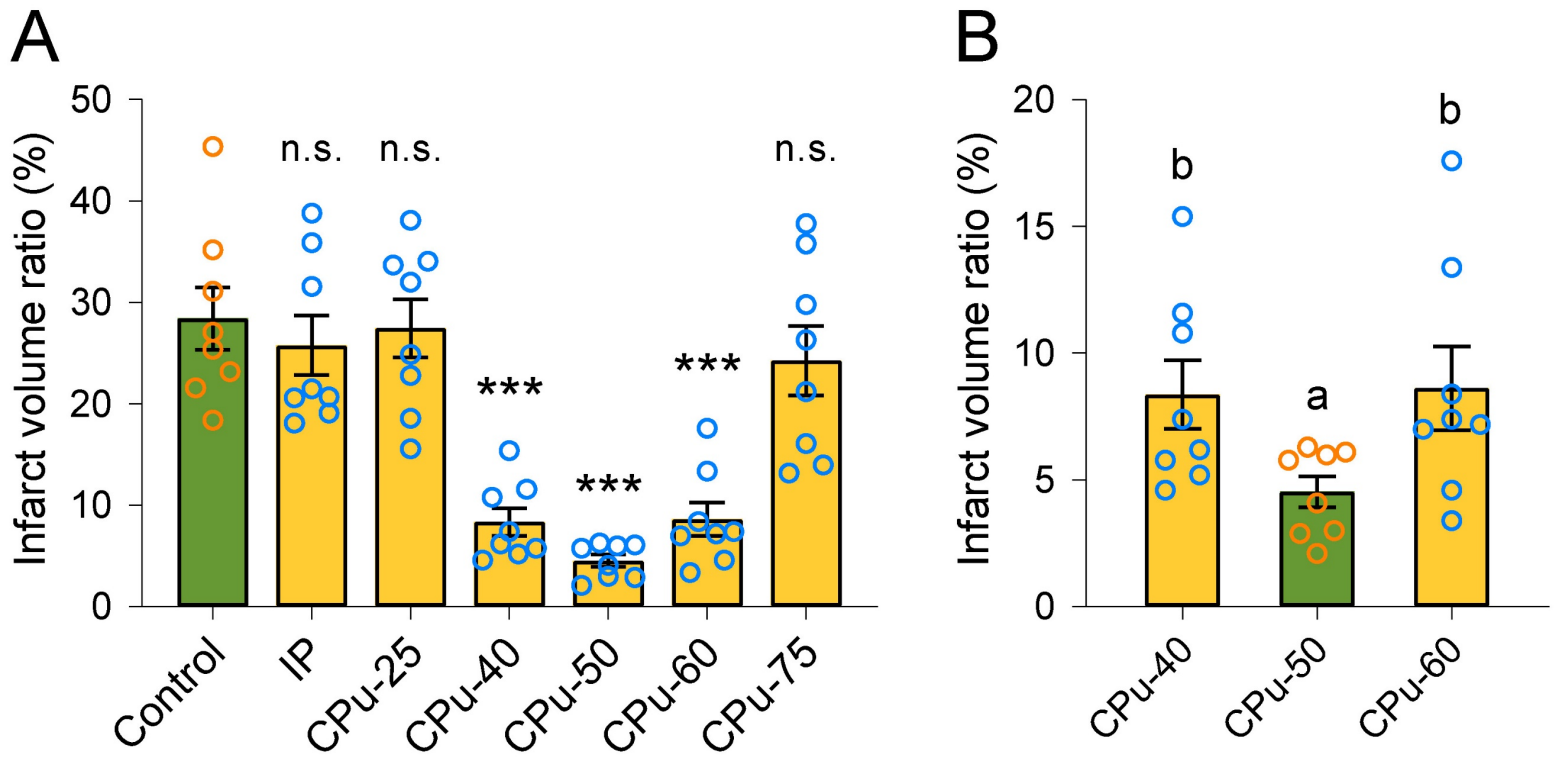
**Brain ECS administration of 40-60 mM citicoline significantly reduces brain infarct volume.** (A) Compared with the control group, the infarct volume ratio of brain slices in the CPu-40, CPu-50, and CPu-60 group was significantly smaller. (B) The infarct volume ratio of brain slices in the CPu-50 group was the smallest among the three groups. Data are expressed as the mean ± standard error. Asterisks indicate significant differences between samples (*, P < 0.05; **, P < 0.01; ***, P < 0.001; Duncan's test; n = 8).

We observed that the infarct volume ratio of rats in the IP group was similar to that in the control group (25.79 ± 4.14% vs 28.43 ± 4.34%, P > 0.05; Duncan's tests; n = 8) (Figure 3A). We also did not detect any remarkable difference in the infarct volume ratio of rats in the CPu-25 group in comparison with that in the control group (27.48 ± 4.07% vs 28.43 ± 4.34%, P > 0.05; Duncan's tests; n = 8) (Figure 3A). Similarly, the infarct volume ratio of rats in the CPu-75 group resembled that in the control group (24.28 ± 4.84% vs 28.43 ± 4.34%, P > 0.05; Duncan's tests; n = 8) (Figure 3A). However, we found that the infarct volume ratio of rats in the CPu-40 group was significant different compared with that in the control group (8.38 ± 1.91% vs 28.43 ± 4.34%, P < 0.001; Duncan's tests; n = 8) (Figure 3A). We also observed that the infarct volume ratio of rats in the CPu-50 group was predominantly different from that in the control group (4.54 ± 0.85% vs 28.43 ± 4.34%, P < 0.001; Duncan's tests; n = 8) (Figure 3A). Finally, the infarct volume ratio of rats in the CPu-60 group was also markedly different from that in the control group (8.63 ± 2.34% vs 28.43 ± 4.34%, P < 0.001; Duncan's tests; n = 8) (Figure 3A).

We also compared the infarct volume ratios between the CPu-40, CPu-50, and CPu-60 groups. We accordingly found that the infarct volume ratio of the CPu-50 group was the smallest among the three groups; however, we did not detect any significant differences between the CPu-40 and CPu-60 groups (P > 0.05; Duncan's tests; n = 8) (Figure 3B).

### CPu-50 group had the smallest neurological deficiency score

**Figure 4:**
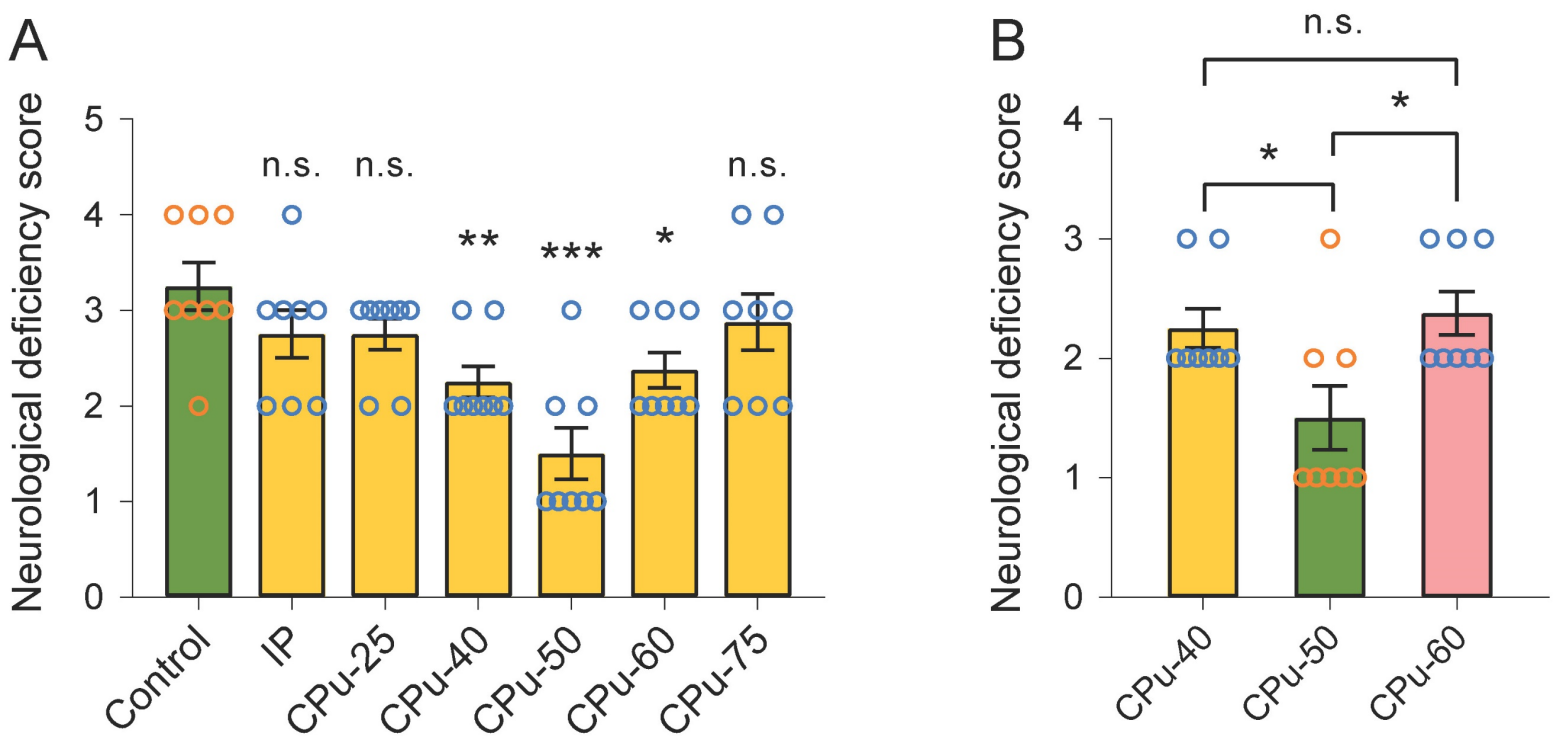
** Brain ECS administration of 40-60 mM citicoline significantly reduces neurological deficiency scores.** (A) Compared with the control group, the neurological deficiency scores of rats in the CPu-40, CPu-50 and CPu-60 group were significantly smaller, respectively. (B) The neurological deficiency scores of rats in the CPu-50 group were the smallest among the three groups. Data are expressed as the mean ± standard error. Asterisks indicate significant differences between samples (*, P < 0.05; **, P < 0.01; ***, P < 0.001; Duncan's test; n = 8).

We did not observe any evident difference in the neurological deficiency scores between the IP and control groups (2.75 ± 0.35 vs 3.25 ± 0.35, P > 0.05; Duncan's tests; n = 8) (Figure 4A). Likewise, the neurological deficiency scores between the CPu-25 and control groups were similar (2.75 ± 0.23 vs 3.25 ± 0.35, P > 0.05; Duncan's tests; n = 8) (Figure 4A). We also noticed that the neurological deficiency score in the CPu-75 group showed no dramatical difference from that in the control group (2.88 ± 0.42 vs 3.25 ± 0.35, P > 0.05; Duncan's tests; n = 8). However, we found that the neurological deficiency score in the CPu-40 group was smaller than that in the control group (2.25 ± 0.23 vs 3.25 ± 0.35, P < 0.01; Duncan's tests; n = 8). In addition, the neurological deficiency score in the CPu-50 group was also smaller than that in the control group (1.50 ± 0.38 vs 3.25 ± 0.35, P < 0.001; Duncan's tests; n = 8); the same occurred in the CPu-60 group (2.38 ± 0.42 vs 3.25 ± 0.35, P < 0.05; Duncan's tests; n = 8).

We further determined that the neurological deficiency score in the CPu-50 group was significantly smaller than that in the CPu-40 (1.50 ± 0.38 vs 2.25 ± 0.23, P < 0.05; Duncan's tests; n = 8) and CPu-60 (1.50 ± 0.38 vs 2.38 ± 0.42, P < 0.05; Duncan's tests; n = 8) groups. However, we did not detect any striking differences in the neurological deficiency scores between the CPu-40 and CPu-60 groups.

## Discussion

In the present study, we demonstrated the efficacy of citicoline against experimental ischemic stroke in rats and obtained the optimal citicoline dose range administered via brain ECS. We found 40-60 mM citicoline administered into the brain ECS dramatically decreased the brain infarct volume in a MCA occlusion rats model. Hence, our study might shed insights into the precise treatment approach when using citicoline for neuroprotection against acute ischemic stroke.

A typical clinical feature of acute ischemic stroke is the rapid onset of neurological impairment, which can be localized in a single cerebral arterial territory 13. Disability and neurological dysfunction caused by acute ischemic stroke lead to a decline in the quality of life of patients and result in severe economic, family, and even the social burdens 14. Therefore, investigating the mechanisms of acute ischemic stroke and identifying novel treatment strategies is of profound significance.

Citicoline or cytidine-5'-diphosphocholine (CDP-choline) is globally used as a dietary supplement or even as a drug 15. Following ingestion or injection in the human body, citicoline is degraded into choline and cytidine, which are salient substrates in neuronal membrane phospholipids biosynthesis 2,15,16. In addition, citicoline can attenuate vascular cognitive impairment in patients with cerebrovascular disease 17. Therefore, the neurovascular protection and repair effects of citicoline might be potentially enhanced in stroke 2. In addition, citicoline administered via the brain ECS pathway prior to a stroke exerted protective effects in a rat madel 4. In this study, we further uncovered that 40-60 mM citicoline administer to the brain ECS may be more effective in treating acute ischemic stroke.

However, due to the heterogeneous nature of human stroke, citicoline may not be efficacious in the treatment of acute ischemic stroke unlike the experimentally induced animal studies of acute stroke 2,18. In addition, the use of citicoline is not recommended for early management in patients with acute ischemic stroke 13,19. As the occurrence of stroke cannot be predicted in clinical practice, the use of citicoline in advance is made impractical. Therefore, clinical evaluation is required to verify the effectiveness of citicoline for treatment of acute ischemic stroke in humans. Nonetheless, after the emergency time of acute ischemic stroke, citicoline may be a good treatment choice.

## Abbreviations

CPu: caudate-putamen
ECS: extracellular space
ISF: interstitial fluid
MRI: Magnetic resonance imaging
IP: intraperitoneal
